# Bipolar button-electrode plasma vaporization of the prostate: An effective option for patients with post-brachytherapy retention

**DOI:** 10.3892/etm.2015.2671

**Published:** 2015-08-05

**Authors:** YIWEI LIN, BEN LIU, LIPING XIE

**Affiliations:** Department of Urology, The First Affiliated Hospital, School of Medicine, Zhejiang University, Hangzhou, Zhejiang 310003, P.R. China

**Keywords:** brachytherapy, prostate cancer, bipolar plasma vaporization of the prostate

## Abstract

Urinary retention is a common urinary complication following brachytherapy for prostate cancer. When conservative therapy has failed, surgical intervention, such as transurethral resection of the prostate (TURP), is performed. In the present case, it was found that conventional bipolar TURP was an inappropriate choice of therapy, since the electrical loop could easily rupture and discharge sparks when encountering the seeds intraoperatively; however, bipolar button-electrode plasma vaporization of the prostate was proven to be a much safer technique. The ‘button-type’ electrode, which has a larger contacting surface, was firm enough to tolerate the transient high levels of energy generated by the short circuit and enable the safe completion of the procedure.

## Introduction

Brachytherapy is an ideal treatment for clinically localized prostate cancer. When compared with radical prostatectomy, brachytherapy is often well-tolerated, with only mild to moderate urinary morbidity; however, urinary complications following brachytherapy, such as bladder-outlet obstruction or urinary retention, have been reported in up to 15% of the men that have undergone the procedure (1). Several alternatives for the treatment of post-brachytherapy retention have been reported in the literature, including prolonged suprapubic catheterization, intermittent self-catheterization, urethral endoprosthesis and transurethral resection of the prostate (TURP) (2,3). The present case report describes the treatment of the condition with bipolar button-electrode plasma vaporization of the prostate, which was found to be a considerably safer procedure than TURP for the management of post-brachytherapy retention.

## Case report

**Figure 1.**
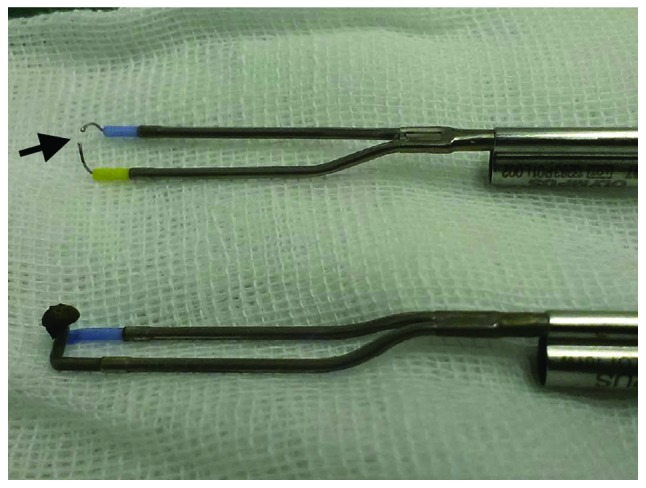
Melted and ruptured electrical loop (arrow) and ‘button-type’ electrode.

**Figure 2.**
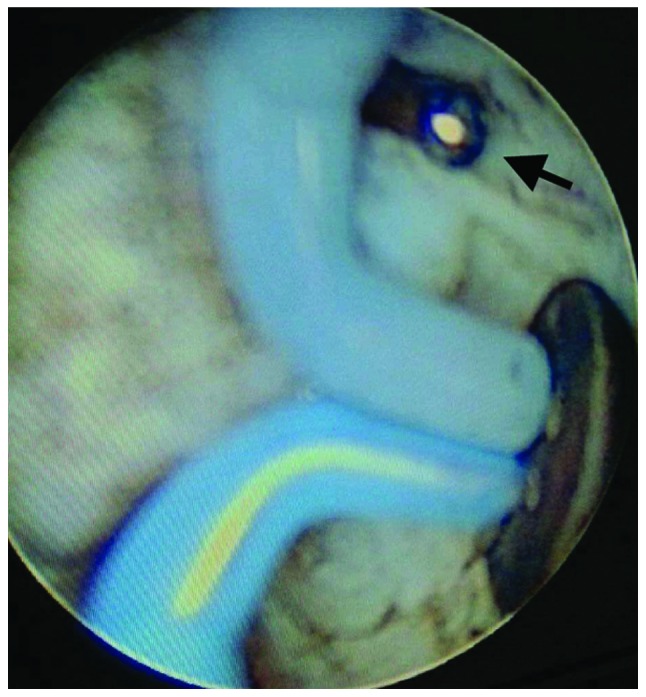
The seed (arrow) was dislodged by the ‘button-type’ electrode.

A 70-year-old male patient with a history of brachytherapy for prostate cancer visited the Department of Urology of the First Affiliated Hospital of the School of Medicine of Zhejiang University (Hangzhou, China) due to urinary retention that had been apparent for 6 months. During those 6 months, the patient had repeatedly failed 5 voiding trials, despite receiving concomitant α-blockers and anti-inflammatory medication. TURP was therefore performed. Written informed consent was obtained from the patient for the publication of this case report. During the bipolar TURP procedure, it was found that the electrical loop generated high levels of energy when encountering the seeds intraoperatively, which, in turn, melted and ruptured the loop (Fig. 1). The electrical loop was thus exchanged for a button electrode, which was used for the vaporization procedure. The ‘button-type’ electrode, which has a larger contacting surface than the electrical loop, could tolerate the high levels of energy generated by the electrode-seed contact well and was firm enough to dislodge the seeds (Fig. 2). The vaporization of the prostate was successfully performed, and the patient recovered uneventfully.

## Discussion

Various improvements and modifications have been made to the surgical treatment of benign prostatic hyperplasia over the last decade. Bipolar technology has gained increasing worldwide popularity, since it allows for the resection of the prostate gland in saline solution. With the use of saline as the irrigant, bipolar TURP reduces the risk of TUR syndrome, which results from hyponatremia following the absorption of irrigation fluid (4). In addition, bipolar resection improves hemostasis, resulting in enhanced intraoperative visualization. Bipolar TURP is therefore preferable as a procedure, due to the fact that it has a more favorable safety profile (5); however, in rare cases, the implanted seeds can short-circuit the conventional electrical loop, which consequently generates high energy levels that can break the electrical loop and cause spark discharge.

A bipolar plasmakinetic vaporization system with a novel ‘button-type’ electrode has been described as a safe and effective alternative for the treatment of patients with lower urinary tract symptoms from bladder-outlet obstruction (6). Compared with conventional endoscopic technique, vaporization (7) or enucleation (8,9) of the prostate with a ‘button-type’ electrode exhibits superior efficacy and safety. In the present case, the bipolar plasmakinetic vaporization system was found to be a more effective surgical instrument than the conventional electrical loop. The button-type electrode has a larger contacting surface, which can disperse the transient high energy levels generated by the short circuit. Furthermore, the button-type electrode is firm enough to dislodge the implanted seeds. We therefore suggest that bipolar plasma vaporization of the prostate with a button electrode is an ideal option for patients suffering from post-brachytherapy retention.
